# Case report: Intraoperative frozen section analysis of Thyroid paraganglioma

**DOI:** 10.3389/fonc.2022.1038076

**Published:** 2022-11-01

**Authors:** Huanyu Zhao, Yudie Lu, Jingrong Zheng, Yuyao Xie, Qingchang Li

**Affiliations:** ^1^ Department of Pathology, The First Hospital and College of Basic Medical Sciences, China Medical University, Shenyang, Liaoning, China; ^2^ Department of Clinical Medicine, The First Clinical College of Jinzhou Medical University, Jinzhou, Liaoning, China

**Keywords:** thyroid paraganglioma, intraoperative frozen section, thyroid follicular adenoma, differential diagnosis, immunohistochemical staining, management approach, case report

## Abstract

Paraganglioma (PGL) is a neuroendocrine tumor that arises from the sympathetic or parasympathetic paraganglia. Primary thyroid PGL is extremely rare. PGL may be difficult to diagnose on frozen sections because its histopathological features, such as polygonal tumor cells with eosinophilic cytoplasm arranged irregularly, overlap with those of thyroid follicular adenoma. We present a case of thyroid PGL in a female patient and provide a detailed description of the patient’s clinicopathologic characteristics. Cervical computed tomography showed a left thyroid mass with uneven density. Intraoperative frozen section analysis showed an uneven fibrous septa and rich networks of delicate vessels surrounding tumor cell nests. The tumor cells were polygonal or epithelioid with eosinophilic cytoplasm, arranged in a nest, trabecular, or organoid pattern were and diagnosed as thyroid follicular adenoma. However, in postoperative immunohistochemistry, these were diagnosed as thyroid PGL. The postoperative recovery was uneventful. The patient showed no signs of tumor recurrence or metastasis until 16 months of follow-up. Herein, we summarize the characteristic features of thyroid PGL based on frozen section analysis. In the appropriate clinical context, its proper use as diagnostic and differential diagnostic management strategies is recommended.

## Introduction

Paraganglioma (PGL) is a neuroendocrine tumor that arises from adrenal or extra-adrenal, sympathetic or parasympathetic paraganglia (1) and accounts for a small fraction of head and neck tumors (2). The most frequent site in head and neck PGL is the carotid body, and primary thyroid PGL is extremely rare. Thyroid PGL is related to the parasympathetic nervous system. Lack of tyrosine hydroxylase results in clinically asymptomatic thyroid PGL. Additionally, thyroid PGL is significantly correlated with mutations in succinate dehydrogenase (3), and a large percentage of hereditary PGL is a result of susceptible gene mutations in the succinate dehydrogenase complex (*SDH*x; including *SDHA*, *SDHB*, *SDHC*, *SDHD*), *TMEM127*, or *MAX* (4). *SDHB* mutations are important because these are strongly associated with the malignant behavior of PGL (5, 6).

Frozen section is used for direct primary diagnosis during surgery and is necessary to avoid inappropriate surgical procedures (7). However, thyroid PGL may be difficult to diagnose on frozen sections owing to its rarity and histopathological features, such as polygonal tumor cells with eosinophilic cytoplasm arranged irregularly, that overlap with those of thyroid follicular adenoma. The histological findings of PGL on frozen sections remain largely unknown. Thyroid PGL is an extremely rare thyroid tumor that is often misdiagnosed as other neoplasms on frozen section analysis. Postoperative paraffin section analysis and immunohistochemical staining are needed to confirm the diagnosis of PGL. Yu et al. described a case of primary thyroid PGL mimicking medullary carcinoma (8, 9). To the best of our knowledge, this report presents the first case of thyroid PGL mimicking follicular adenoma on preoperative imaging and intraoperative frozen section. We aimed to recommend a strategy for the diagnosis and differential diagnosis of thyroid PGL.

## Case description

### Clinical presentation

A 33-year-old Chinese woman was first admitted to the hospital in August 2020. The patient presented with an egg-sized tumor (4 cm in diameter) in the left side of her neck and had been found to have a thyroid nodule on routine examination (color ultrasonography) 4 years prior. The patient had no other significant medical history. Before hospitalization, the patient did not experience pain, hoarseness, dysphagia, panic, dyspnea, or agitation, and the patient’s diet, defecation, and weight were normal.

Hospital examination revealed consistent, bilateral thoracic respiratory movements. The following signs were not observed: ankle clonus, Brudzinski’s sign, Babinski’s sign, Hoffmann’s sign, and asterixis. No enlarged systemic superficial lymph nodes were found, and no tumor was found in the breast. The vital signs were normal (body temperature, 36.6°C; blood pressure, 111/63 mmHg; pulse, 92 beats/min; respiration, 20 breaths/min).

### Laboratory investigations

Ultrasonography showed a 6.5 cm × 4.6 cm × 3.5 cm inhomogeneous nodule with solid echogenicity in the left thyroid gland and a remarkable surrounding hypervascular flow signal. Thyroid function test results were unremarkable, and serum calcium levels were within the normal range. Preoperative serum calcitonin and thyroglobulin levels were 1.55 pmol/L (normal range: 0.00–5.22 pmol/L) and 9.38 ng/mL (normal range: 1.6–59.9 ng/mL), respectively.

**Figure 1:**
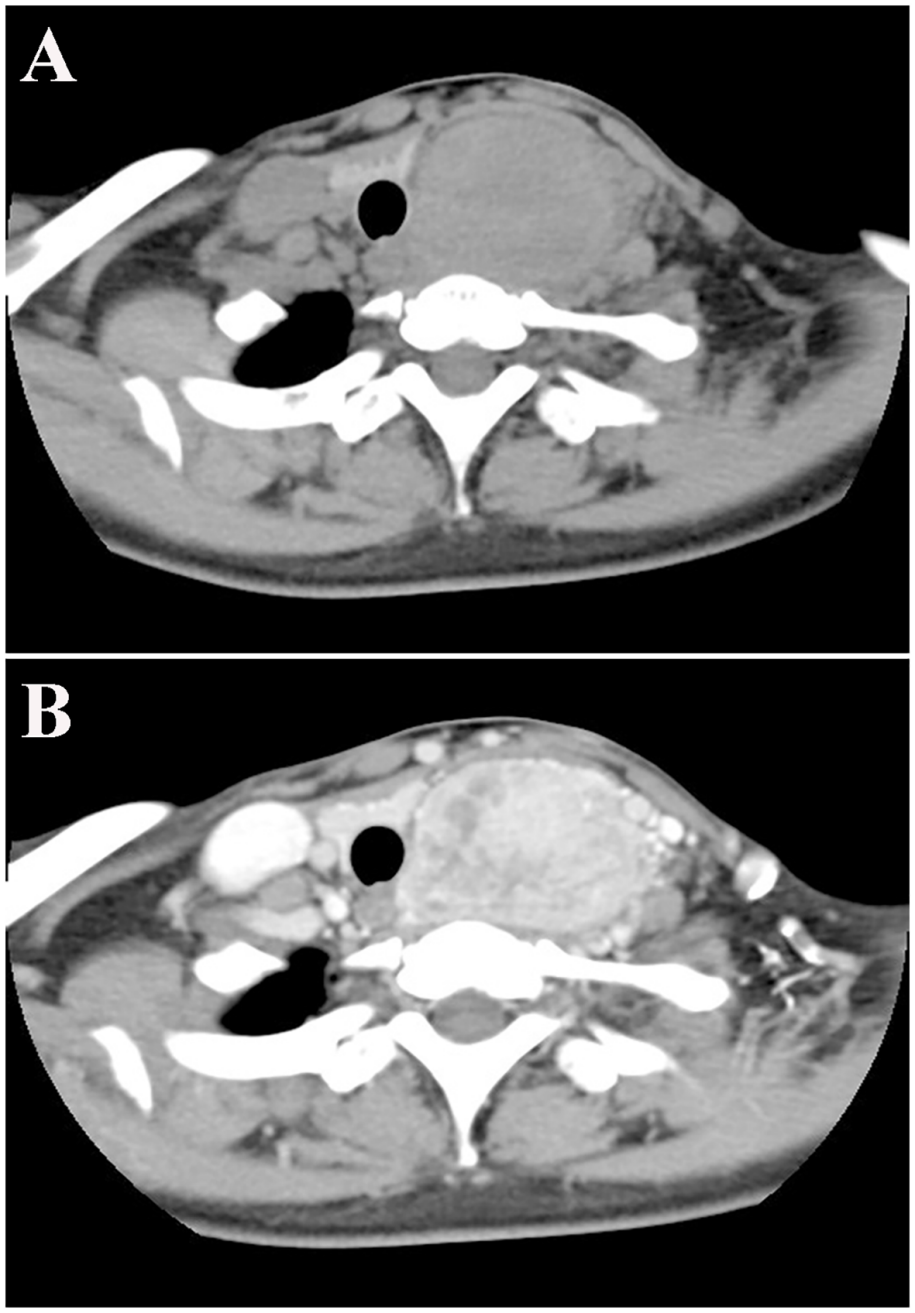
Cervical computed tomography scan **(A, B)**.

Cervical computed tomography (CT) showed a left thyroid mass (diameter: 5.5 cm) with uneven density and a plain CT value of 63 Hounsfield units (HU) (**Figure 1A**), suggestive of an adenoma. The trachea was compressed and deviated to the right. An enhanced scan showed multiple blood vessels in a heterogeneously enhanced lesion with a CT value of 133 HU (**Figure 1B**). No abnormal cervical lymphadenopathy was detected.

### Pathological examination with a light microscope

The thyroid mass was clinically considered a benign tumor, and subtotal thyroid lobectomy was performed. The poorly demarcated tumor was located in the posterior left lobe and grossly measured 6.5 cm × 4.5 cm × 3 cm. The resected section was grayish red and had moderate consistency.

**Figure 2:**
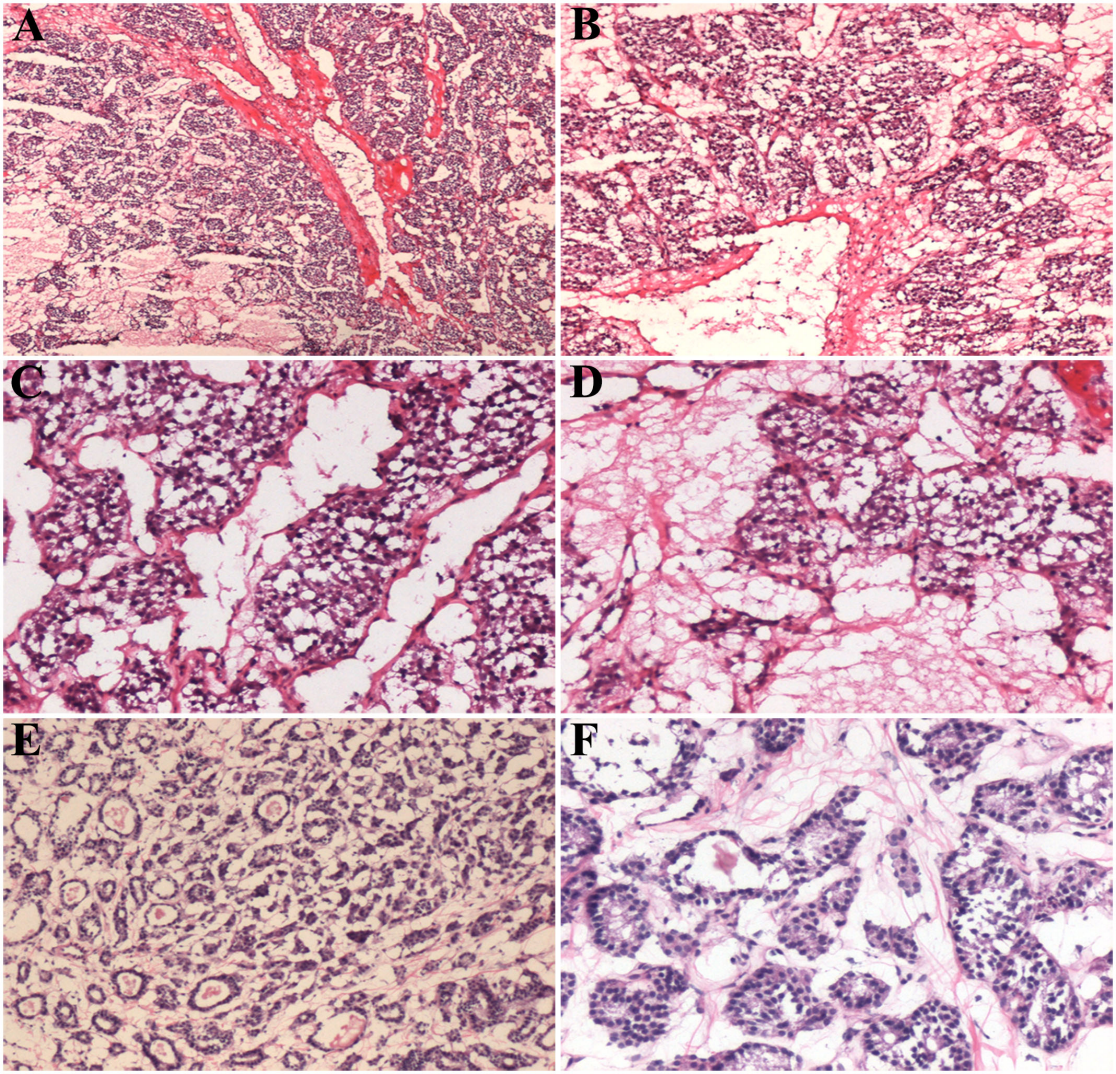
Histological features of the intraoperative frozen sections of thyroid paraganglioma **(A–D)** and follicular adenoma **(E, F)**. Scale bars: 400 μm **(A, B)**, 200 μm **(C, D)**, 300 μm **(E)**, and 100 μm **(F)**.

Intraoperative frozen section showed an uneven fibrous septa and rich networks of delicate vessels surrounding tumor cell nests (**Figures 2A, B**). The interstitial fibers were extensively hyalinized. The tumor tissue comprised polygonal or epithelioid cells with eosinophilic cytoplasm arranged in a nesting, trabecular, or organoid pattern (**Figures 2C, D**). Enlarged tumor cells with hyperchromatic nuclei were scattered in the lesion. Frozen section examination initially supported the diagnosis of the nodule as thyroid follicular adenoma.

### Differential diagnosis

Follicular adenoma is characterized by follicular cells arranged in a microfollicular, solid, or trabecular pattern. Its cytologic features and architectural pattern are different from those of a normal thyroid follicle, with the absence of nuclear grooves and ground glass nuclei. Follicular adenoma shows a mild, atypical, organizational structure comprising tumor cells (**Figures 2E, F**). In PGL, the cell nests are closely arranged with a rich, vascular, invasion-like blood vessel network.

**Figure 3:**
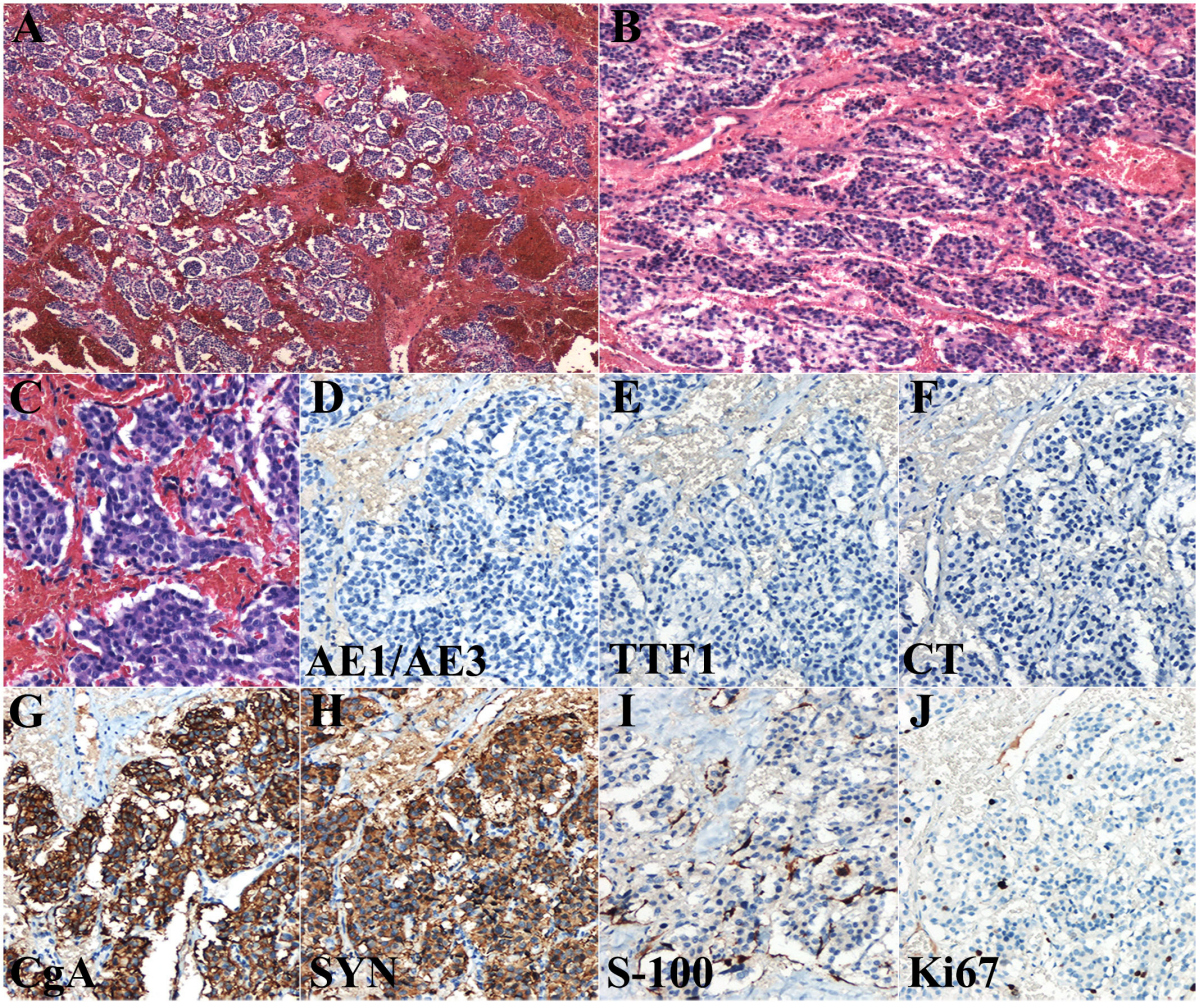
Histological features of the postoperative paraffin section **(A–C)** and immunohistochemical examination **(D–J)** of thyroid paraganglioma. Scale bars: 400 μm **(A)**, 300 μm **(B)**, and 100 μm **(C–J)**. CgA, chromogranin A; CT, calcitonin; Syn, synaptophysin.

Postoperative paraffin sections are shown in **Figures 3A-C**. The cytoplasm of the cells were eosinophilic, and the nuclei were slightly atypical. The cell nests were either strip shaped or mass like with rich vascular networks. In addition, hyaline degeneration was observed between the vascular networks. These characteristics were in concordance with those of thyroid PGL.

Immunohistochemical staining was performed for auxiliary diagnosis. The tumor cells were negative for AE1/AE3, TTF1, and calcitonin (**Figures 3D-F**), and positive for chromogranin A and synaptophysin (**Figures 3G, H**). Chromogranin A and synaptophysin are markers of neuroendocrine tumors. These immunohistochemical findings support the diagnosis of thyroid PGL. S-100 protein staining showed elongated sustentacular cells at the periphery of the tumor cell nests (**Figure 3I**). The Ki67 index was approximately 7% (**Figure 3J**).

The morphological characteristics of the intraoperative frozen section are not typical for the differential diagnosis of intrathyroidal PGL. However, the morphological pattern of PGL cannot be distinguished from other neoplasms of different lineage, such as medullary thyroid carcinoma and hyalinizing trabecular tumor (PGL-like adenomas) of the thyroid gland. Therefore, using immunohistochemical markers (**Table 1**) is important for the differential diagnosis of intrathyroidal PGL with lesions.

**Table 1:** Differential diagnosis of intrathyroidal PGL with the other lesions by immunohistochemistry.

Thyroid lesion	Immunostaining pos	Immunostaining neg
PGL	CgA, Syn, NSE, GATA3	TTF1, Tg, CT, CK
Medullary thyroid carcinoma	TTF1, CT, CEA, CgA, *PAX8* ^1^	Tg, CK19
Follicular adenoma	Tg, TTF1	Gal-3, HBME-1, CgA, Syn
Hyalinizing trabecular tumor	Tg, TTF1, Ki67 (MIB1)*	CT
Parathyroid tissue	CK, CgA, Syn, PTH, GATA3	Tg, TTF1
Metastasis	PSA^2^, AR^2^, CD10^3^, CK7^4^, SP-A^4^	TTF1^5^

AR, androgen receptor; CD10, common acute lymphocytic leukemia antigen; CD56, neural cell adhesion molecule; CEA, carcinoembryonic antigen; CgA, chromogranin A; CT, calcitonin; CK, cytokeratin; Gal-3, galectin-3; HBME-1, human bone marrow endothelial cell marker-1; Neg, negative; NSE, neuron-specific enolase; PAX-8, paired-box gene 8; PGL, paraganglioma; Pos, positive; PSA, prostate specific androgen; PTH, parathyroid hormone; SP-A, surfactant protein A; Syn, synaptophysin; Tg, thyroglobulin; TTF1, thyroid transcription factor 1.; SP-A, surfactant protein A.

^1^Wweak focal expression, ^2^originated from prostate adenocarcinoma, ^3^originated from clear-cell renal carcinoma, ^4^originated from lung adenocarcinoma, ^5^originated from lung adenocarcinoma and positive for, TTF1 positive.

*Ki-67 cell membrane reactivity is unique in the hyalinizing trabecular tumor but occurs only when the MIB1 monoclonal antibody is used at room temperature (10, 11).

The patient did not undergo post-surgical radiotherapy or chemotherapy. Subsequent postoperative investigations, including thyroid function tests and serum calcium levels, cervical ultrasonography, and CT of the neck and chest, were normal. After 16 months of follow-up, the patient was alive with no signs of tumor recurrence or metastasis. The timeline with relevant data from the episode of care is presented in **Figure S1**.

## Discussion

Thyroid PGL is an uncommon neuroendocrine tumor. Generally, PGL is underrecognized due to its rare occurrence. To date, no reported cases have been correctly diagnosed by preoperative imaging examination. Frozen section analysis is a vital aspect during surgery (7); however, thyroid PGL is seldom diagnosed correctly through this method. Therefore, frozen section analysis is necessary for the histological diagnosis of thyroid PGL and as a differential diagnostic approach.

The differential diagnoses of thyroid PGL on frozen section includes medullary thyroid carcinoma, follicular neoplasm, metastatic renal cell carcinoma, metastatic neuroendocrine tumor, hyalinizing trabecular tumor, and parathyroid proliferation. Histologically, thyroid PGL comprises two types of cells: polygonal chief cells and long, narrow sustentacular cells. Tumor cells grow as a distinctive organoid or nesting structure around delicate vessels with prominent stromal sclerosis. In PGL, tumor cell nests are closely arranged with rich, vascular, invasive blood vessel networks.

According to previous reports, most patients with thyroid PGL present with a neck mass, and other symptoms are rare (12, 13). Almost no correct diagnoses have been made based on frozen sections. Most cases were diagnosed as malignant based on the histological characteristics of solid cell nests composed of slightly pleomorphic nuclei (8). In the present case, an incorrect diagnosis was made based on the frozen section analysis of follicular adenoma. Thyroid adenoma can lie within the thyroid parenchyma, where neoplastic cells grow in a solid, trabecular, nodular, or follicular pattern and the stroma is sparse and richly vascularized. The final diagnosis of thyroid PGL was confirmed by paraffin section and immunohistochemical analyses.

Frozen section analysis is essential to guide surgical procedure; however, this was not applicable in the present case. Thyroid PGL is seldom diagnosed correctly by frozen section analysis. In the future, we will collect frozen sections from as many cases of thyroid PGL as possible, in order to summarize their histological characteristics. Additionally, the diagnostic approach for detecting thyroid nodules generally involves ultrasonography and cytology. Due to the lack of typical morphological characteristics on intraoperative frozen section, thyroid PGL must be diagnosed through preoperative fine-needle aspiration cytology with intraoperative frozen section analysis.

The treatments for thyroid PGL and follicular adenoma have several differences. As a benign thyroid tumor, follicular adenoma is treated *via* total or subtotal thyroid lobectomy (14). Head and neck PGLs have a low metastatic potential (15). Malignant metastases can occur in areas where paraganglionic tissue is not normally present, such as the lymph nodes. Hence, surgical resection combined with radiotherapy could be necessary in some cases of thyroid PGL (16, 17). For unresectable tumors, radioactive isotope treatment and chemotherapy may be helpful (18). In our case, the patient did not receive radiotherapy after surgery.

Pheochromocytoma and PGL are usually hereditary. Muth et al. recommends that all patients with pheochromocytoma and PGL should undergo genetic screening for the detection of germ line variants of genes, such as *FH*, *NF1*, *RET*, *SDHB*, *SDHD*, and *VHL*, regardless of patient and family characteristics. In addition, the genetic testing of *MEN1*, *SDHA*, *SDHAF2*, *SDHC*, *TMEM127*, and *MAX* genes is recommended (19). The loss of immunoreactivity for SDHB is associated with mutations in *SDHA*, *SDHB*, *SDHC*, *SDHD*, and *SDHAF2*; therefore, germline mutation testing of *SDHB*, *SDHC*, and *SDHD* is recommended in patients with SDHB-negative tumors (20). Overall, we believe that genetic screening will become an important auxiliary diagnostic strategy for patients with thyroid PGL in the future.

In our report, we summarized the characteristic features of thyroid PGL based on frozen section analysis, which had a differential diagnostic significance. These features are as follows: (a) severe interstitial fibrosis with edema and hyperemia, (b) cuboidal cells containing hyperchromatic nuclei and growing in strip- or organ-like morphology with irregular shape and size, and (c) vascularized tissues encircling the tumor cell mass. Further advances in PGL diagnosis are expected in the future. However, we examined only one case, and data from similar cases must be collected to summarize more meaningful characteristic pathologic findings on frozen sections. Our patient did not have typical symptoms, and PGL was not considered by the clinician, leading to a misdiagnosis on frozen section. Therefore, patients with thyroid PGL must be examined by using meaningful imaging features and performing genetic and laboratory testing.

PGL is a neuroendocrine tumor derived from the sympathetic or parasympathetic paraganglia. Primary thyroid PGL is extremely rare. This case emphasized that PGL can present in uncommon locations without lymphadenopathy or unremarkable blood test results. Intraoperative frozen histopathology in thyroid PGL has features that overlap with those of follicular adenoma, such as polygonal tumor cells with eosinophilic cytoplasm arranged irregularly. Thyroid PGL is characterized by closely arranged cell nests surrounded by a highly vascular network. The characteristic finding of thyroid PGL was evident in our case. These features are necessary for the diagnosis and differential diagnosis of thyroid PGL. Taking note of these will avert a misdiagnosis.

## Patient perspective

I am a 33-year-old woman. A few years ago, a mass was found in my left neck on physical examination. Recently, I was able to feel it myself, so I decided to undergo surgical resection. Based on my radiology test results, my physician considered the mass benign. During the operation, a frozen section was performed, and the pathologist diagnosed it as thyroid follicular adenoma. The surgeon performed subtotal thyroid lobectomy; however, postoperative pathology diagnosed my mass as thyroid PGL. Fortunately, the doctor did not suggest a second surgery. After the wound healed, I had an uneventful discharge. Re-examination after 3 months showed that my surgical site had recovered well, and laboratory test results, such as those for thyroid function, were also in the normal range.

## Data availability statement

The original contributions presented in the study are included in the article/**Supplementary Material**. Further inquiries can be directed to the corresponding author.

## Ethics statement

Written informed consent was obtained from the individual(s) for the publication of any potentially identifiable images or data included in this article.

## Author contributions

HZ, JZ, YL, and YX collected data and wrote the paper. HZ and QL revised the manuscript. All authors contributed to the article and approved the submitted version.

## Funding

This study was supported by the National Natural Science Foundation of China (grant number 81602022) and the Department of Science and Technology of Liaoning Province (grant number 2021-MS-197).

## Acknowledgments

We would like to thank Weinan Li for their technical support.

## Conflict of interest

The authors declare that the research was conducted in the absence of any commercial or financial relationships that could be construed as a potential conflict of interest.

## Publisher’s note

All claims expressed in this article are solely those of the authors and do not necessarily represent those of their affiliated organizations, or those of the publisher, the editors and the reviewers. Any product that may be evaluated in this article, or claim that may be made by its manufacturer, is not guaranteed or endorsed by the publisher.
