# Supplementary figures and images for: Case report: Intraoperative frozen section analysis of Thyroid paraganglioma
Source: Front Oncol. 2022 Nov 1;12:1038076. doi: 10.3389/fonc.2022.1038076 (PMC9664199; doi:10.3389/fonc.2022.1038076)

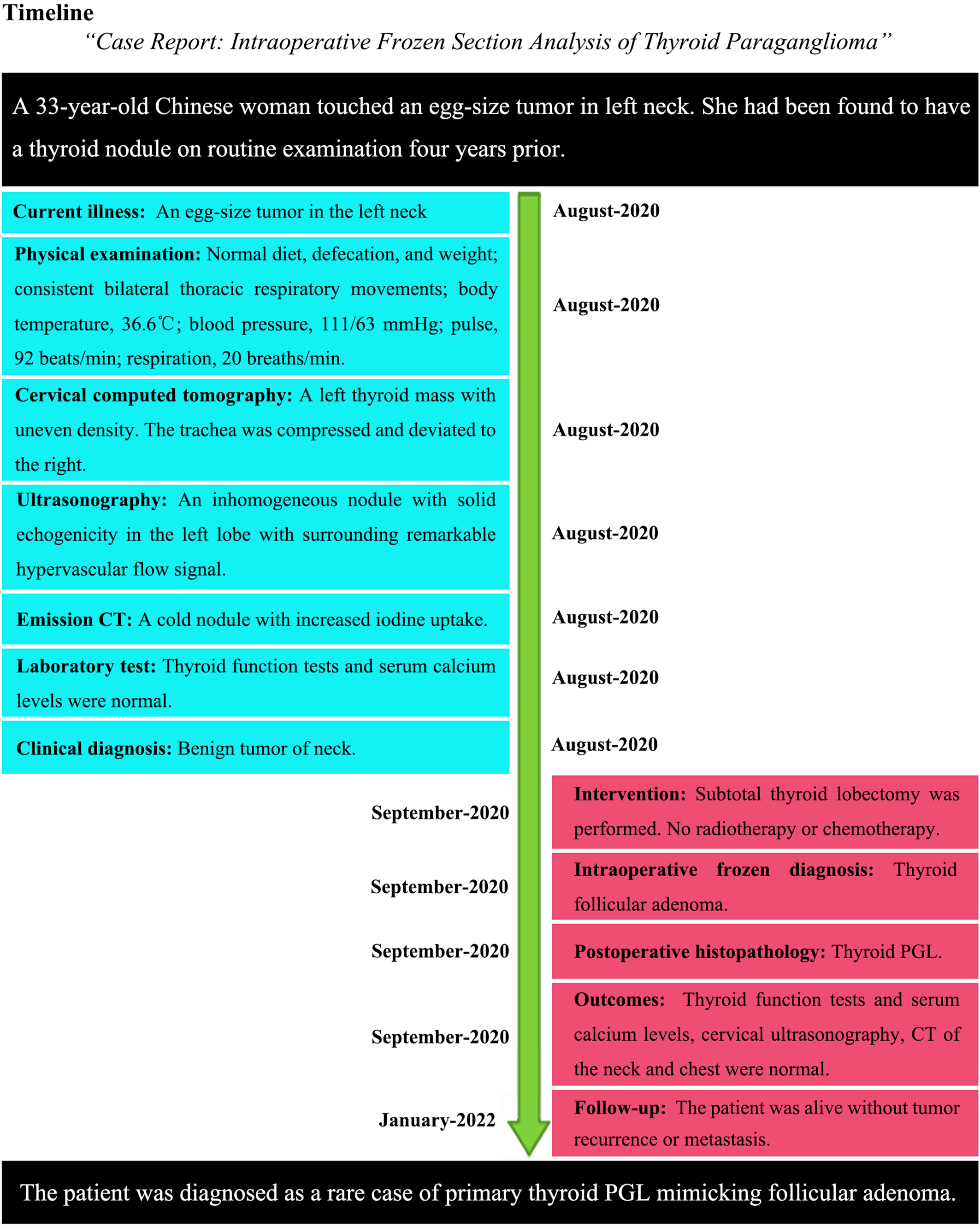

Supplement: Supplementary file 1 [file Image_1.tif]
